# Recombinant Flagellin-Porcine Circovirus Type 2 Cap Fusion Protein Promotes Protective Immune Responses in Mice

**DOI:** 10.1371/journal.pone.0129617

**Published:** 2015-06-12

**Authors:** Chunyan Zhang, Shanshan Zhu, Li Wei, Xu Yan, Jing Wang, Rong Quan, Ruiping She, Fengjiao Hu, Jue Liu

**Affiliations:** 1 Beijing Key Laboratory for Prevention and Control of Infectious Diseases in Livestock and Poultry, Institute of Animal Husbandry and Veterinary Medicine, Beijing Academy of Agriculture and Forestry Sciences, No. 9 Shuguang Garden Middle Road, Haidian District, Beijing 100097, People’s Republic of China; 2 College of Veterinary Medicine, China Agricultural University, No. 2 Yuanmingyuan West Road, Haidian District, Beijing 100197, People’s Republic of China; Federal University of São Paulo, BRAZIL

## Abstract

The Cap protein of porcine circovirus type 2 (PCV2) that serves as a major host-protective immunogen was used to develop recombinant vaccines for control of PCV2-associated diseases. Growing research data have demonstrated the high effectiveness of flagellin as an adjuvant for humoral and cellular immune responses. Here, a recombinant protein was designed by fusing a modified version of bacterial flagellin to PCV2 Cap protein and expressed in a baculovirus system. When administered without adjuvant to BALB/c mice, the flagellin-Cap fusion protein elicited stronger PCV2-specific IgG antibody response, higher neutralizing antibody levels, milder histopathological changes and lower viremia, as well as higher secretion of cytokines such as TNF-α and IFN-γ that conferred better protection against virus challenge than those in the recombinant Cap alone-inoculated mice. These results suggest that the recombinant Cap protein when fused to flagellin could elicit better humoral and cellular immune responses against PCV2 infection in a mouse model, thereby acting as an attractive candidate vaccine for control of the PCV2-associated diseases.

## Introduction

Porcine circovirus type (PCV), within the family *Circoviridae*, consists of two genotypes. PCV1 is considered to be non-pathogenic to pigs [1], whereas PCV2 is the major causative agent of postweaning multisystemic wasting syndrome (PMWS) [2], and other clinical disorders including porcine dermatitis and nephropathy syndrome, reproductive failure, acute pulmonary edema, as well as growth retardation, which are collectively referred to as PCV2-associated diseases (PCVAD) [2–4]. Severe PCV2 infection may hijack immune system followed by inducing immunosuppression in pigs, leading to an increased susceptibility to secondary infections as well as reduced immune responses to vaccinations against other infectious diseases [5]. PCVAD is now recognized as serious threats to the swine industry worldwide.

Two major open reading frames (ORFs) have been identified for PCV, ORF1, called *rep* gene, which encodes a protein of 35.7 kDa associated with virus replication [6], and ORF2, called *cap* gene, which encodes the host-protective immunogenic capsid protein of 27.8 kDa [7,8]. Use of vaccines has become the most cost-effective strategy for protecting pigs against PCV2 infection. To date, several kinds of PCV2 vaccine development have been reported, most of them based upon the expression of the PCV2 capsid protein in bacterial vector [9,10], baculovirus expression vector [11–13], Trichoplusia ni-larvae [14], and live-vectored expression systems [15–17]. Baculovirus expression system has been widely used to express heterologous genes in cultured insect cells. Baculovirus-derived recombinant proteins are expressed at high concentrations and maintain both their natural antigenicity and immunogenicity. There are two currently available commercial PCV2 vaccines which are based on baculovirus-expressed recombinant Cap protein produced in insect cell-based bioreactors [18,19]. Both induction of humoral and cellular immunity has been shown to promote protection against PCV2 infection [20]. Therefore, it would be interesting to find novel measures to enhance the efficacy of recombinant Cap protein vaccines when administered to animals.

Toll-like receptors (TLRs) are an evolutionarily conserved type of pattern recognition receptors that modulate host immune response via binding conserved structural motifs on pathogens. Flagellin, the major structural component of the flagellar filament of gram-negative bacteria, is the ligand for TLR5 and it can promote activation of dendritic cells and other cells including macrophages, endothelial cells, and epithelial cells [21–24], and the production of chemokines and cytokines including TNF-α and IFN-γ that help to initiate and modulate the adaptive immune response [24]. Flagellin has been extensively used as a potent systemic and mucosal adjuvant in vaccine development [25–30]. Administration with flagellin-antigen fusion proteins are capable of producing enhanced protective responses such as remarkably high titers of antigen-specific IgG and secretion of critical cytokines and chemokines when compared to antigen proteins alone. In the present study, a recombinant PCV2 Cap protein fused with flagellin was expressed in baculovirus expression system and the recombinant flagellin-Cap fusion protein was used to further evaluate its vaccine potential in terms of immunogenicity and protection against PCV2 challenge in BALB/c mice.

## Materials and Methods

### Ethics Statement

The present study was approved and performed in accordance with the animal welfare guidelines (IACUC-2010) of the Animal Care and Use Committee of Institute of Animal Husbandry and Veterinary Medicine Beijing Academy of Agriculture and Forestry Sciences. All efforts were made to minimize suffering of animals.

### Cells and viruses

Spodoptera frugiperda clone 9 (Sf9) cells, purchased from Invitrogen, were grown and maintained in monolayer cultures at 27°C using Sf-900 II SFM media (Invitrogen) supplemented with 100 U/ml of penicillin G, and 100 μg/ml streptomycin. Recombinant strains of Autographa Clifornica nuclear polyhedrosis virus were propagated in Sf9 cells. For PCV2 infection, PK15 cells, purchased from ATCC, were grown in monolayer cultures and infected with PCV2 strain BJW [31] at a multiplicity of infection (MOI) of 1 50% tissue culture infective dose (TCID_50_) and additionally treated with 300 mM D-glucosamine at 20–24 h after inoculation as described previously [32].

### Cloning and expression of recombinant flagellin-Cap protein

Recombinant flagellin fused with PCV2 Cap protein was expressed according to the protocol of the Bac-to-Bac Baculovirus Expression System (Invitrogen). The flagellin from *Salmonella typhimurium* fljb was modified as described previously [33] and the sequence of the resultant flagellin with a length of 810 bp was synthesized. The resultant flagellin fused to Cap gene of PCV2 strain BJW [31], a full-length of 1,509 bp, was cloned into vector PUC57 to obtain recombinant plasmid PUC57-flagellin-Cap and stored in our laboratory. The flagellin-Cap fusion gene was amplified by polymerase chain reaction (PCR) using one pair of oligonucleotide primers from plasmid PUC57-flagellin-Cap. The pair of primer sequences for this fusion gene amplification were F: 5’- CCCTCGAGGATGGCACAAGTAATCAACACTAAC-3’ and R: 5’- CCCAAGCTTGGAGGGTTAAGTGGGGGGTCTTTAA-3’. To facilitate cloning of the PCR fragment, XhoI and HindIII restriction enzyme sites (underlined) were incorporated into the forward (F) and reverse (R) primer sequences, respectively. They were subsequently cloned into the pFastBacHTa vector under the control of the polyhedral promoter. The resultant pFastBacHTa recombinant vector was transformed into DH10Bac competent cells by site-specific transposition with Tn7 to obtain a bacmid carrying the flagellin-Cap fusion gene. The recombinant bacmid was then transfected into Sf9 cell monolayers grown in six-well cell culture plates to produce recombinant baculovirus. Expression of recombinant protein was confirmed by an immunofluorescence assay (IFA) using rabbit anti-Cap polyclonal antibody.

### Indirect immunofluorescence and confocal microscopy

Sf9 monolayer cells seeded in chamber slides were infected with recombinant baculovirus. At the indicated times, the cells were washed with phosphate-buffered saline (PBS) and fixed in 4% paraformaldehyde (PFA). After three washes, the cells were incubated with rabbit anti-Cap antibody, which was derived from sera of rabbits after immunization with recombinant Cap protein three times, diluted in 3% bovine serum albumin (BSA)-PBS at room temperature (RT) for 1 h. After three further washes, cells were incubated with fluorescein isothiocyanate (FITC)-conjugated anti-rabbit antibodies (DAKO) at 37°C for 1 h. Nuclei were visualized by staining with 4’,6’-diaminido-2-phenylindole (DAPI) at a concentration of 1 μg/ml for 30 min at 37°C. Cells were washed three times with PBS, rinsed in dH_2_O, dried and mounted with fluorescence mounting media, and examined under Nikon AIR confocal laser microscope system.

### Production and purification of recombinant protein

The viral titer of the recombinant flagellin-Cap baculovirus was assayed and expressed as plaque forming unit (PFU) according to the standard protocol. Sf9 cells were infected with the recombinant virus followed by assaying protein expression at different harvest time intervals. The optimized harvesting time was 72 h postinfection. For protein production, Sf9 cells were infected with the recombinant baculovirus at a MOI of 1 PFU and harvested at the indicated time point. The expressed fusion protein was purified under denaturing conditions using the ProBond Purification System (Invitrogen, USA) in accordance with the manufacturer’s protocol. Briefly, the recombinant baculovirus-infected Sf9 cells for 72 h after infection were collected and centrifuged at 3,000 × g for 35 min. The cell lysates were resuspended in 8 ml of 6.0 M guanidine hydrochloride (pH 8.0) and applied to a ProBond Nickel-Chelating Resin with a His-tag for purification of recombinant fusion proteins. Prior to inoculation into mice, the purified proteins under denaturing conditions were further dialyzed against 0.5 M NaCl in 50 mM phosphaste buffered solution (PBS) (pH 7.4) with a step-wise reduction of urea concentrations. As a control, Sf9 cells were infected with a recombinant Cap baculovirus (35) and the purified recombinant Cap protein was included in animal experiments.

### Western-blotting

For immunoblotting analysis, recombinant proteins resolved by 10% sodium dodecyl sulfate-polyacrylamide gel electrophoresis (SDS-PAGE) were transferred onto nitrocellulose membrane with a mini Trans-Blot transfer electrophoresis unit (Bio-Rad). The membranes were blocked for 2 h at RT in TBST blocking buffer (20 mM Tris-HCl [pH 7.4], 150 mM NaCl, 0.1% Tween 20) containing 5% skim milk powder to prevent nonspecific binding and then incubated with specific polyclonal primary antibodies raised against Cap protein, flagellin (Invitrogen), β-actin (Santa Cruz), or monoclonal antibody against histidine tag (Invitrogen) at RT for 2 h. The membranes were washed three times with TBST buffer and incubated for 2 h at RT with horseradish peroxidase (HRP)-conjugated secondary antibodies diluted in blocking buffer. Immunoreactive bands were visualized by enhanced chemiluminescence system (Kodak Image Station 4000R).

### Immunization of BALB/c mice with recombinant flagellin-Cap protein

A total of 36 8-week-old, specific-pathogen–free female BALB/c mice were purchased from Vital River Laboratories, Beijing, China. The mice were randomly allocated six groups (6 mice of each group) and housed in filter-top cages in isolation rooms under controlled temperature (21–23°C), humidity (30–70%), and lighting (14 hours light/10 hours dark) and fed sterilized food and water *ad libitum*. In groups 1 and 2, the mice were injected intramuscularly at both hind legs with 2 μg of recombinant Cap and flagellin-Cap proteins dissolved in 100 μl of PBS and boosted once at 2 weeks interval, respectively. As groups 1 and 2 immunization protocol, the mice in groups 3 and 4 were administered in a volume of 100 μl (10 μg of recombinant Cap and flagellin-Cap proteins) and boosted once at 2 weeks interval, respectively. Groups 5 and 6 were set as negative control and sham inoculated with 100 μl of PBS. Serum samples were collected weekly after primary inoculation to detect Cap protein-specific ELISA antibodies and neutralizing antibodies against PCV2. Two weeks after booster, all of the mice in groups 1 through 5 were inoculated intraperitoneally with the PCV2 strain BJW at 10^5^ TCID_50_ in a total volume of 100 μl that harvested from infected PK15 cells. The mice in group 6 served as non-PCV2 challenged control. The animals were monitored daily for mortality, weight loss, and presence of any clinical symptoms after PCV2 challenge. Three weeks after challenge, serum samples were collected for determining the presence of PCV2 DNA, and all the mice were humanely euthanized via cervical dislocation following isoflurane anesthesia, and their inguinal lymph nodes were collected for pathological analysis. All sections of this study follow the ARRIVE Guidelines for reporting animal research [34]. A Completed ARRIVE guidelines checklist is shown in S1 Checklist.

### Enzyme-linked immunosorbent assay (ELISA)

Polystyrene microtiter plates (Nunc, Denmark) were coated with the purified recombinant Cap protein (50 ng per well) in 50 mM carbonate buffer (pH 9.6) overnight at 4°C. After absorption, the plates were washed three times with TBST and blocked with blocking buffer (5% skimmed milk powder in TBST) at 37°C for 30 min. Serum samples from the mice were diluted 1:50 in diluting buffer (diluted 1% skimmed milk powder in TBST) and incubated for 60 min at 7°C. After washing three times with TBST, plates were treated with HRP-conjugated goat anti-mouse immunoglobulin G (Sigma) diluted 1:8 000 in diluting buffer with 1% skimmed milk powder for 60 min at 37°C. The reaction was developed by using 3,3,5,5-tetramethylbenzidine (TMB) as the chromogen. The OD value of each well was measured at 450 nm using an ELISA reader. Serum samples with an optical density greater than those of normal mouse sera (means ± three standard deviations) were considered seropositive for PCV2.

### Serum neutralization assay

The PCV2 neutralization capability of mouse antisera raised against recombinant flagellin-Cap and Cap proteins was determined by using an end-point dilution reduction assay. Briefly, serum samples collected from each time point after recombinant vaccinations were inactivated at 56°C for 30 min followed by serial dilution in 2-fold increments in Dulbecco’s modified Eagle medium (DMEM) and incubated with an equal volume of 200 TCID_50_ of PCV2 strain BJW. After incubation for 60 min at 37°C, the antibody–virus mixtures were inoculated onto monolayer PK15 cells in a 96-well plate. After 60 min of incubation at 37°C, the inoculum was removed, cell cultures were washed twice with DMEM and culture medium was added for incubation. Each dilution was inoculated into four wells. After 72 hours of incubation, cells were fixed and incubated with rabbit anti-Cap antibody followed by fluorescein isothiocyanate (FITC)-conjugated anti-mouse immunoglobulin G (Sigma). By fluorescence microscopy, it was observed at which concentration of the serum, PCV2-positive cells could still be detected. The neutralization titer was calculated as the reciprocal of the highest serum dilution that was able to completely block PCV2 infection in PK15 cells.

### Cytokine levels

Cytokine levels, tumor necrosis factor (TNF)-α and interferon (IFN)-γ were measured by respective mouse ELISA kit obtained from Invitrogen according to the protocols of the manufacturer. Briefly, serum samples collected at the indicated time points of BALB/c mice after inoculation of recombinant proteins were treated with Standard Diluent Buffer and used for cytokine quantification by ELISA.

### Quantitative real-time polymerase chain reaction (PCR)

Quantitative real-time PCR was performed to determine PCV2 DNA levels in the mouse serum samples collected at three weeks after PCV2 challenge. Total DNA was extracted from 10 μl of serum by using the QIAamp DNA blood minikit according to the manufacturer’s instructions. A forward primer (5’-ATCAAGCGAACCACAG-3’) and a reverse primer (5’-GGTCATAGGTGAGGGGC-3’) were used to amplify a 139-bp fragment from the ORF2 of PCV2. The real-time PCR protocol followed the instructions of a iQ SYBR green Supermix kit (Bio-Rad). The PCR parameters are comprised of total denaturation at 95°C for 5 min and 40 cycles of denaturation at 95°C for 10 s and annealing at 55°C for 10 s. For a standard curve, serial dilutions of plasmid pFastBac-Cap were used to quantify the virus genomic copy number. The geometric numbers of viral genomic copies for each sample were expressed as the mean value of duplicate reactions. The threshold sensitivity of this assay was 1 copy per microliter.

### Pathological examination

Samples of inguinal lymph nodes were collected and fixed by immersion in 2.5% glutaraldehyde-polyoxymethylene solution. Fixed tissues were dehydrated, embedded in paraffin wax, sectioned at 4 mm thickness, and then stained with hematoxylin and eosin (HE) for light microscopic observation. As previously described [35], inguinal lymph node changes were estimated as an amount of infrequent apoptotic cells, lymphoid depletion and histiocytic infiltration and/or the presence multinucleated cells ranging from 0 (normal) to 3 (severe apoptotic changes, lymphoid depletion and/or histiocytic infiltration), and the score of microscopic lesions were counted in six fields of view.

### Statistical analysis

Results are presented as averages ± the standard deviation (SD) of the means, as indicated. Statistical comparisons are made by using Student’s *t* test, and differences between groups were considered significant if the P value was less than 0.05.

## Results

### Expression of recombinant flagellin-Cap fusion protein

**Fig 1:**
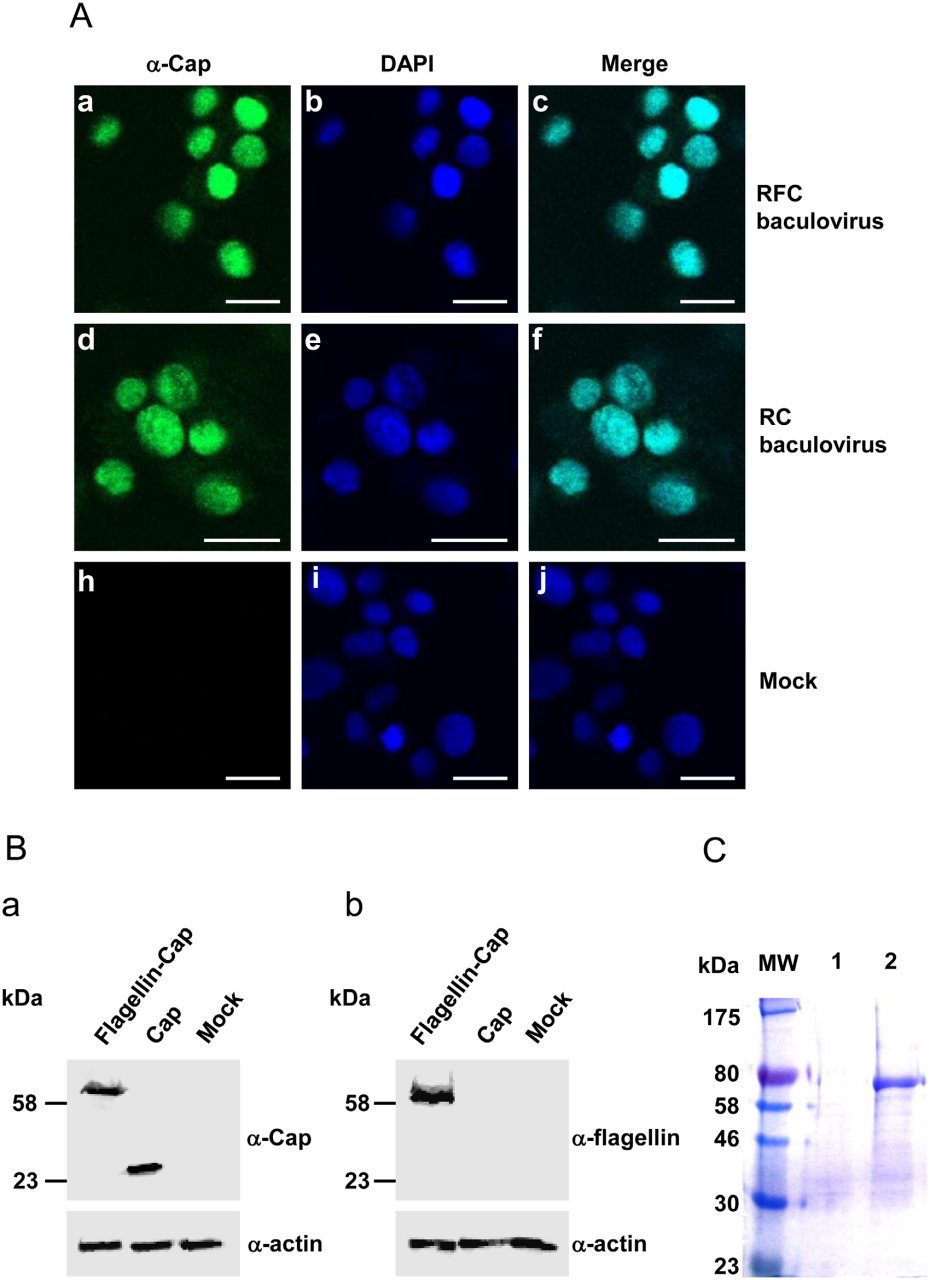
Expression of the flagellin-Cap fusion protein in baculovirus system. (A) Immunofluorescence analysis. Sf9 cells were infected with the recombinant flagellin-Cap baculovirus and recombinant Cap baculovirus, fixed at 72 h postinfection and incubated with rabbit polyclonal antibody raised against PCV2 Cap protein, followed by DAPI staining for nucleic localization. Bars, 20 μm. (B) Sf9 cells infected with the recombinant flagellin-Cap (RFC) and Cap (RC) baculoviruses were electrophoresed in a 10% SDS-PAGE gel and transferred onto a nitrocellulose membrane for Western-blotting. Transferred proteins were incubated with rabbit antibody against Cap protein (a) or mouse antibody against flagellin (b) followed by incubation with HRP conjugated IgG secondary antibodies. Mock-infected Sf9 cells were included as a negative control, and anti-β-actin was used as a loading control of protein extracts. (C). SDS-PAGE of the purified flagellin-Cap fusion protein. Lane W, molecular weight markers; lane 1, mock; lane 2, 2.5 μg flagellin-Cap fusion protein.

The flagellin-Cap fusion fragment was cloned into the Bac-to-Bac donor plasmid followed by being transposed into the bacmid genome to construct a recombinant bacmid. The recombinant bacmid carrying the flagellin-Cap fragment was transfected into Sf9 cells to obtain a recombinant baculovirus. The Sf9 cells were infected with the recombinant baculovirus at an MOI of 1 PFU for protein expression. Expression of recombinant flagellin-Cap fusion protein was determined by the IFA using a rabbit polyclonal antibody raised against PCV2 Cap protein or a mouse monoclonal antibody against His-tag and was found to localize in the nuclei of Sf9 cells (Fig 1A and data not shown). As observed for localization of Cap protein in the recombinant Cap baculovirus-Sf9 cells (Fig 1A), fusion to the flagellin protein did not change the Cap localization. Furthermore, total cell lysates from recombinant flagellin-Cap baculovirus infection were subjected to 10% SDS-PAGE and followed by Western blotting for detecting expression of the recombinant protein. The recombinant flagellin-Cap fusion protein had a size of 61 kDa (Fig 1B, panel a), which correlates to its expected size including the 5 kDa N-terminal His6-tag. The expression of flagellin was also detected by Western blotting (Fig 1B, panel b). In addition, no signals were detected in mock-infected Sf9 cells when reacted with anti-Cap antibody or anti-flagellin antibody on Western blotting (Fig 1B, panels a and b). The expressed recombinant protein was purified under denature conditions using Ni-NTA resin, subjected furthermore to electrophoresis (Fig 1C) and shown subsequently to react specifically with anti-Cap antibody (data not shown).

### Serum antibody against recombinant flagellin-Cap protein

**Fig 2:**
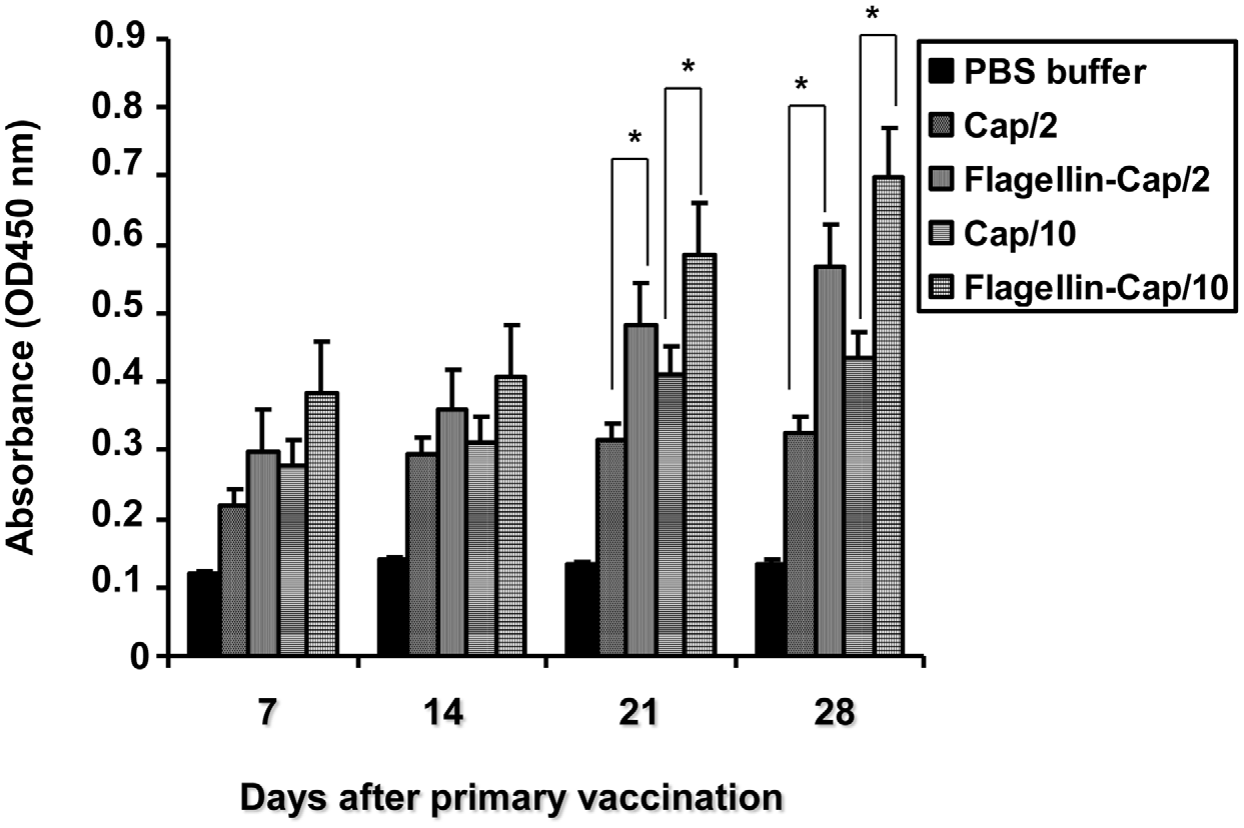
Kinetics of PCV2 Cap-specific antibody in mice vaccinated with the flagellin-Cap fusion and Cap protein at various times post-vaccination by using an indirect ELISA. Results represent mean absorbance values of each group sera samples ± SD from two independent experiments. Significant difference is expressed as *p*<0.05 (*) for a comparison of the flagellin-Cap fusion and Cap protein-vaccinated mice at the indicated time points.

The titers for serum antibody against PCV2 Cap protein in the vaccinated mice were determined using an indirect ELISA (Table 1). All PBS buffer-inoculated mice were negative for anti-PCV2 antibody throughout the study. In group 1 mice that were vaccinated with Cap protein (2 μg), seroconversion to antibody of PCV2, was first detected at 14 days post-vaccination (dpv) with 1 of 6 mice, followed by 21 dpv with 3 of 6 mice, and 28 dpv with 4 of 6 mice, had seroconverted to PCV2. In group 2 mice that were vaccinated with the flagellin-Cap fusion protein (2 μg), seroconvesion to PCV2 Cap-specific antibody first occurred at 7 dpv with 3 of 6 mice, but by 14 dpv, all of the rest mice had seroconverted to PCV2 Cap-specific antibody. In group 3 mice that were vaccinated with Cap protein (10 μg), seroconversion to antibody of PCV2 was first detected at 7 dpv with 1 of 6 mice, followed by 14 dpv with 3 of 6 mice, but by 21 dpv, all of the rest mice had seroconverted to PCV2 Cap-specific antibody. In group 4 mice that were vaccinated with the flagellin-Cap fusion protein (10 μg), all the mice had seroconverted to PCV2 Cap-specific antibody at 7 dpv and retained seroconversion overtime. PCV2 Cap-specific antibody dynamics during the experiment are shown in Fig 2. The titers of PCV2-specific antibodies in the mice that were vaccinated with the flagellin-Cap fusion protein were higher than those vaccinated with the Cap protein from 7 to 28 dpv, when administered with the same doses. Furthermore, the flagellin-Cap fusion protein protocols induced PCV2 Cap-specific antibody was dose-dependent. These data clearly showed that the flagellin-Cap fusion protein elicited higher antibody levels in ELISA than the Cap protein alone.

**Table 1:** Seroconversion to PCV2 antibodies in mice vaccinated with flagellin-Cap fusion proteins.

Group	Vaccination	No. of mice with PCV2 antibodies/no. tested^a^ at dpv			
		7	14	21	28
1	Cap/2	0/6	1/6	3/6	4/6
2	Flagellin-Cap/2	3/6	6/6	6/6	6/6
3	Cap/10	1/6	3/6	6/6	6/6
4	Flagellin-Cap/10	6/6	6/6	6/6	6/6
5	PBS buffer	0/6	0/6	0/6	0/6

^a^Sera of six mice from each group were collected at different time points after vaccination.

### Neutralizing antibody

**Fig 3:**
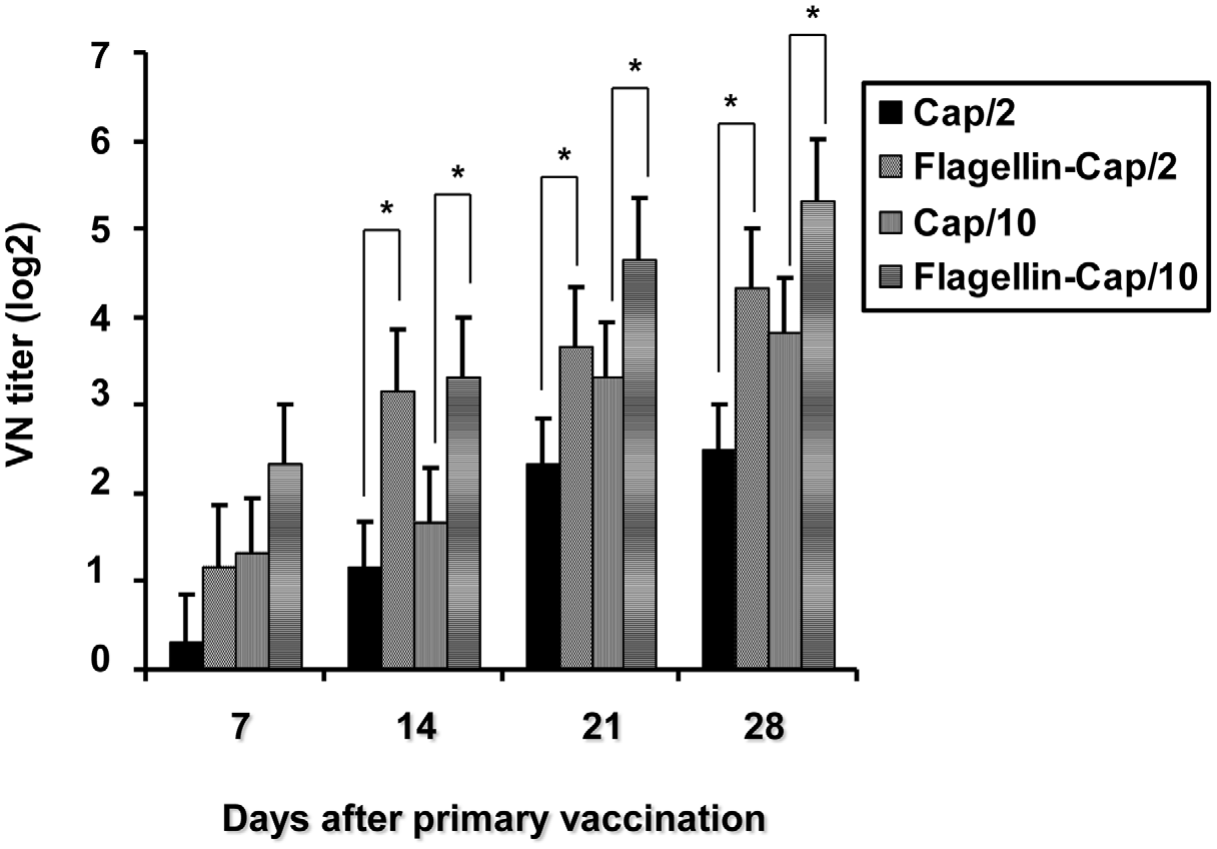
PCV2-neutralization antibody responses after vaccination with the flagellin-Cap fusion and Cap protein. Serum samples from the flagellin-Cap fusion and Cap protein-vaccinated groups at various times after vaccination were used to determine PCV2-neutraliztion antibody titers by using an end-point dilution reduction assay. Results represent mean absorbance values of each group sera samples ± SD from two independent experiments. Significant difference is expressed as *p*<0.05 (*) for a comparison of the flagellin-Cap fusion and Cap protein-vaccinated mice at the indicated time points.

We further determined PCV2 neutralizing antibody titers of serum samples collected from each time point after vaccination. Prior to vaccination, no detectable neutralizing antibodies were observed in mice in any group (data not shown). As shown in Fig 3, serum neutralizing antibody titers to PCV2 in the mice that were vaccinated with the Cap protein alone were undetectable until day 21 post-vaccination (with an antibody titer being 1:5.67) which increased thereafter, regardless of the amounts of the administered doses. The neutralizing antibody titer in the flagellin-Cap fusion protein group 2 was positive at 14 dpv (with an antibody titer being 1:9) and steadily increased thereafter. For group 4, the neutralizing antibody titer was positive at 7 dpv followed by a statistically rise thereafter, with a titer being 1:40 at 28 dpv. No detectable antibody was observed in the mock-vaccinated mice. These results showed that the protocol using the flagellin-Cap fusion protein was more effective than that with the Cap protein alone in inducing PCV2-neutralizing antibody responses in mice.

### Upregulation of TNF-α and IFN-γ induced by the flagellin-Cap fusion protein

**Fig 4:**
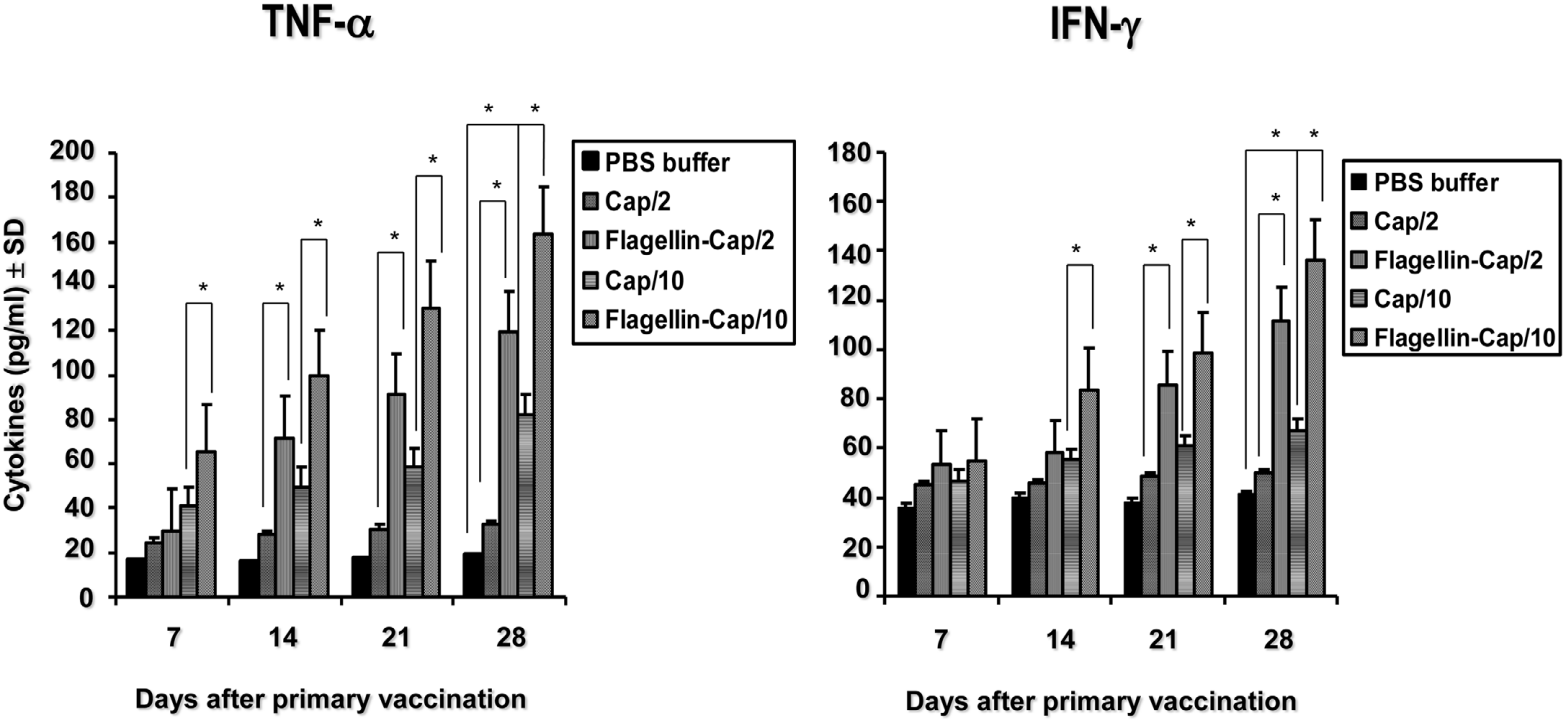
TNF-α and IFN-γ levels in serum of the flagellin-Cap fusion and Cap protein-vaccinated mice. Serum samples from the flagellin-Cap fusion and Cap protein-vaccinated groups at various times after vaccination were used to quantify TNF-α (A) and IFN-γ (B) by enzyme-linked immunosorbent assay. Results represent mean absorbance values of each group sera samples ± SD from two independent experiments. Significant difference is expressed as *p*<0.05 (*) for a comparison of the flagellin-Cap fusion and Cap protein-vaccinated mice, or of the Cap protein-vaccinated and non-vaccinated mice at the indicated time points.

Flagellin, an agonist for TLR5, has been shown to upregulate the levels of some cytokines including TNF-α and IFN-γ for enhancement of cellular immunity. To further determine the levels of TNF-α and IFN-γ induced by the flagellin-Cap protein, we analyzed the production of these two cytokines in the serum of mice. As shown in Fig 4, the levels of TNF-α and IFN-γ in mice of the flagellin-Cap fusion protein-vaccinated groups 2 and 4 at 14 through 28 days after primary vaccination were statistically higher than those in group 1 and 3 mice, respectively. Furthermore, the level of TNF-α in group 4 mice at 7 days dpv was significantly higher than those in group 3 mice. For the Cap protein-vaccinated groups, only group 3 mice at 28 days but not other time points exhibited significantly higher levels of TNF-α and IFN-γ (P<0.05) as compared to those in the non-vaccinated control mice. These results indicated that the flagellin-Cap fusion protein is superior to the Cap protein alone in inducing TNF-α and IFN-γ expression.

### Pathological examination and evaluation of viremia

**Fig 5:**
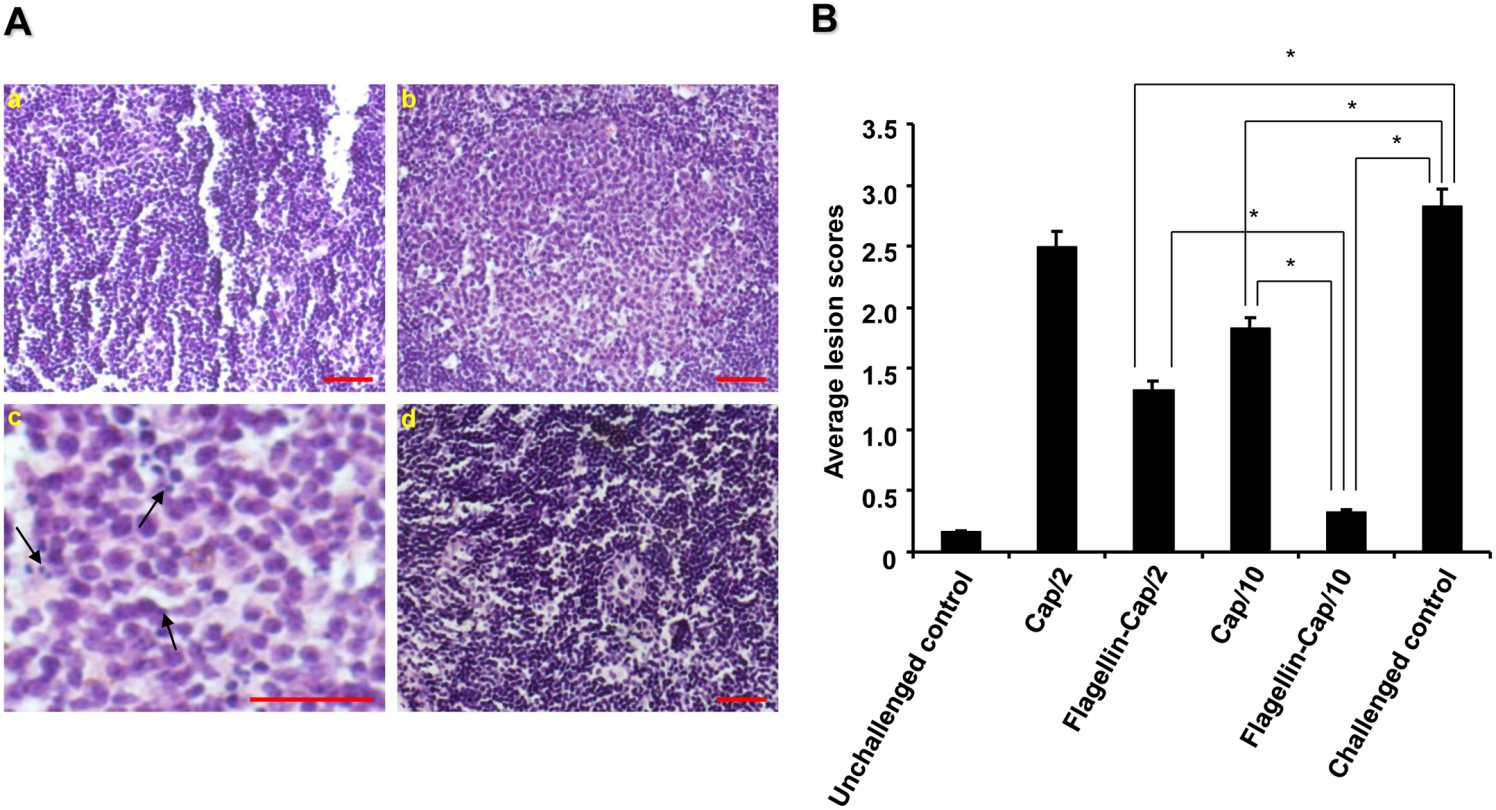
Histopathologic lesions in the inguinal lymph nodes of the flagellin-Cap fusion protein-vaccinated mice after PCV2 challenge. (A) Mice were challenged with PCV2 strain BJW at 2 weeks after booster and the presence of lymph node lesions was examined at 3 weeks after challenge. (a) Normal morphology of inguinal lymph node section from an unvaccinated unchallenged mouse shows no noticeable germinal centers. (b) Infiltration of histiocytes, depletion of lymphocytes, and apoptotic cells were seen in the prominent germinal centers of inguinal lymph node of an unvaccinated PCV2-challenged mouse. (c) Partial enlargement of panel b, showing large numbers of apoptotic cells (arrows) in germinal centers. (d) No obvious lesions were seen in the inguinal lymph node of a recombinant flagellin-Cap fusion protein-vaccinated PCV2-challenged mouse (10 μg dose). Bars, 80 μm. (B) Histopatholoical lesions were evaluated as a score of 0 to 3 in the inguinal lymph nodes of all the mice after PCV2 challenge. The data show the mean scores of six mice in each group from one of two independently repetitive experiments. Significant difference is expressed as *p*<0.05 (*) for a comparison of the flagellin-Cap fusion and Cap protein-vaccinated mice, or of the recombinant protein-vaccinated and non-vaccinated mice after PCV2 challenge.

To investigate the protective efficacy of the flagellin-Cap fusion protein, we compared clinical symptoms and pathological changes in the challenged mice. No clinical signs or gross lesions were observed in the PCV2-inoculated mice at any time after challenge. Inguinal lymph nodes were further collected from all control and inoculated mice at 21 days post-challenge (dpc) for microscopic observation. No microscopic lesions were detected in the non-PCV2 challenged control group 6 mice. PCV2-inoculated unvaccinated group 5 mice exhibited prominent germinal centers that occupied most of the lymph node parenchyma, with infrequent apoptotic cells, lymphocyte depletion, and histiocytic infiltration (Fig 5A). In contrast, no or mild lymphoid depletion and histiocytic replacement (Fig 5A and data not shown) were observed in the inguinal lymph nodes of the vaccinated PCV2-inoculated mice, with the flagellin-Cap fusion protein-vaccinated group 4 mice at the dose of 10 μg being no microscopic lesions. Mean scores of microscopic lesions in the lymph nodes in all the immunized mice but group 1 mice were significantly different (P<0.05) from those of the unvaccinated PCV2-challenged group mice (Fig 5B). In addition, the mean scores of lesions in mice from the flagellin-Cap fusion protein-vaccinated group 4 was statistically different (P<0.05) from those in group 2 and 3 mice (Fig 5B).

In addition to histopathological evaluation, we also quantified serum viral DNA loads of all PCV2-inoculated mice using real-time PCR (Table 2). Non-PCV2 challenged control animals (group 6) were negative for PCV2 viremia. All of the challenged control mice (group 5) were positive for PCV2 viraemia in serum sample with the mean numbers of genomic copies being 5.8 ± 1.2 log PCV2 load copies per 1 ml. Compared with the challenged control mice, PCV2 viremia was detected in all six mice in group 1, three of six mice in group 2, three of six mice in group 3, and only one of six in group 4. Group1 mice showed no obvious decreased PCV2 viremia (5.1 ± 1.6 log PCV2 load copies per 1 ml). But all the three other vaccinated groups mice exhibited significant reductions in the levels of viremia (P<0.05) after PCV2 challenge, with the mean numbers of genomic copies being 3.6 ± 1.5, 4.6 ± 1.8, and 3.2 log PCV2 load copies per 1 ml for groups 2, 3, and 4, respectively. Among the different vaccination protocols, group 1 mice vaccinated with the Cap protein exhibited the highest PCV2 loads, whilst group 4 mice with the flagellin-Cap fusion protein showed the lowest PCV2 loads.

**Table 2:** Viral DNA load in mouse serum measured by real-time PCR.

Group	No. of mice with viremia/no. tested (mean log PCV2 load ± SD) after PCV2 challenge^a^
Cap/2	6/6 (5.1 ± 1.6)
Flagellin-Cap/2	3/6 (3.6 ± 1.5) *
Cap/10	3/6 (4.6 ± 1.8)*
Flagellin-Cap/10	1/6 (3.2)*
Unchallenged control	0/6 (0)
Challenged control	6/6 (5.8 ± 1.2)

^a^Asterisks within columns represent significantly different PCV2 loads between the flagellin-Cap fusion or Cap protein-vaccinated and non-vaccinated challenged mice after challenge (*p*0.05). A load value of 0 is equal to 1 000 copy per milliliter.

Taken together, these results clearly indicated that the flagellin-Cap fusion protein-vaccinated mice exhibited milder histopathological changes and lower viremia than those in the Cap protein alone after PCV2 challenge.

## Discussion

PCVAD is now recognized as a major problem of economic importance in many pig-rearing countries and has become a serious threat to the pig industry worldwide. Immunization against PCV2 infection has been demonstrated to be a remarkably effective means for control of PCVAD occurrence. The Cap protein is the primary target for vaccination against PCV2 infection. Currently, there are two commercially available PCV2 vaccines based upon recombinant Cap protein in baculovirus expression system [18,19]. Although mice may not be an ideal animal model to resemble PCV2 infection as observed for pigs [36], PCV2 can infect and replicate in some mouse strains including BALB/c mouse when used with the appropriate inoculating dose and administered route [35,37]. Therefore, the mouse model has been used for evaluating the immunogenicity and protection of PCV2 vaccines [11,14,38–40]. In the present study, we used the BALB/c mouse model for evaluating the immunogenicity and protective capabilities of an experimental vaccine based on the recombinant flagellin-Cap fusion protein expressed in the baculovirus system. Our results clearly demonstrated that the flagellin-Cap fusion protein elicited significantly higher humoral and cellular mediated immune responses, lower virus loads in serum, as well as lower histopathological scores that afford better protection against PCV2 challenge as compared to that in the Cap protein alone.

Research data have shown that PCV2-specifc antibodies are associated with protection, as evidenced by contribution of the reduced antibodies to the development of PCVAD [41,42]. Further research suggested that the level of neutralization antibody conferred protection against PCV2 infection [39]. In the present study, the mice vaccinated with the flagellin-Cap fusion protein exhibited higher titers of serum antibody than that in the Cap protein alone (Fig 2). Consistent with the PCV2-specific antibody, the mice vaccinated with the flagellin-Cap fusion protein induced higher titers of neutralization antibody than that in the Cap protein alone (Fig 3). In the case of PCV2-specific neutralization antibody, some researches reported that recombinant adenovirus expressing the Cap protein generated VN antibody titers ranging from 1:8 to 1:17 in mice [40] and all plasmids encoding Cap protein with different subcellular localizations induced VN antibody titers of <1:10 in mice [11], but other research reported that no VN antibody against PCV2 was detected in the immunized mice regardless of the pORF2, pORF2/Cap, or Cap/pORF2 vaccinations [39]. Before PCV2 challenge, we found that the mice vaccinated with the flagellin-Cap fusion protein at the dose of 10 μg developed the highest titer of VN antibody (1:40), capable of inhibiting PCV2 infection, with 5 of 6 vaccinated animals being PCV2 negative by real-time PCR at 21 days post-challenge, implying that the vaccine induces good protection immunity. Therefore, our results also demonstrated that the Cap-specific neutralizing antibody plays an important role in protecting against PCV2 infection. However, a standard method for determining the titer of VN antibody against PCV2 needs to be established in the future.

Flagellin can stimulate innate immune responses via its binding to TLR5, thereby promoting the establishment of subsequent humoral and cell-mediated adaptive immunity [30,43,44]. The fusion of flagellin to viral or bacterial proteins, such as West Nile virus [33], influenza virus [45], poxvirus [46], and *Yersinia pestis* [47], was found to enhance the efficacy of vaccines. The results presented here suggest that flagellin enhanced cell-mediated responses and the production of inflammatory molecules as evidenced by higher upregulation of TNF-α and IFN-γ, respectively, as compared to the Cap protein alone (Fig 4). TNF-α is a pleiotropic cytokine that promotes dendritic cell maturation [23] and flagellin-stimulated TNF-α production appeared to enhance the antibody response [48]. CD4^+^ T cells mediate the killing of organisms responsible for a variety of intracellular infections through the production of IFN-γ [49,50]. Research evidences have shown that the sole induction of a humoral response might not guarantee full protection against PCV2 infection, and cell-mediated immune response with the development of PCV2-specific IFN-γ might contribute together with neutralizing antibodies to viral clearance and help avoid progression of PCV2 infection [18,20,51–53]. Therefore, for PCV2 subunit vaccines, the addition of some adjuvant which might mediate cellular immune response will facilitate to promoting protection against PCV2 infection, because IFN-γ responses induced by the Cap protein are lower than with the whole virus [20]. The fusion of flagellin to Cap protein significantly upregulates the IFN-γ response (Fig 4), thereby mediating strong cellular immune response, this may implicate that the recombinant fusion protein confers a better protective response against PCV2 infection.

Flagellin has served as a potent adjuvant in a broad range of model-system vaccination regimens [54,55]. Prior immunity to flagellin does not influence the role of flagellin as an effective adjuvant for eliciting robust responses [48,56]. Previous studies [14,39] demonstrated that the Cap protein conferred a partial protective response required 35 μg or 100 μg of Cap protein with three times administration in mice. In contrast, the flagellin-Cap fusion protein elicited sufficient protection from PCV2 challenge without the use of adjuvant and at significantly lower protein doses (10 μg of protein delivered twice) in the present study. The enhanced efficacy of the flagellin-Cap protein as an immunogen is attributed to the fusion of Cap to flagellin. This may be related to that the high-affinity association of flagellin with TLR5 enhances cellular activation and the uptake of the associated antigen by TLR-positive dendritic cells [57,58]. Therefore, the flagellin-Cap fusion protein is clearly of practical important and might become an alternative cost-effective subunit vaccine against PCV2 infection due to its good immunogenicity and efficacy when administered at a low dose in the absence of an adjuvant.

## Conclusions

By linking the Cap protein to flagellin, we have generated a novel fusion protein capable of eliciting a strong PCV2 specific immune response that is efficacious against PCV2 infection. On the basis of its immunogenicity and efficacy in the mouse model here, we believe that this recombinant fusion protein holds great promise as a PCV2 vaccine in pigs and that this approach will provide new opportunities for use in a broad range of recombinant protein-based vaccines against other infectious diseases in pigs.

## Supporting Information

S1 Checklist “The ARRIVE Guidelines Checklist” for reporting animal data in the present manuscript. (DOC)
