# Supplementary material for: Recombinant Flagellin-Porcine Circovirus Type 2 Cap Fusion Protein Promotes Protective Immune Responses in Mice
Source: PLoS One. 2015 Jun 12;10(6):e0129617. doi: 10.1371/journal.pone.0129617 (PMC4466504; doi:10.1371/journal.pone.0129617)

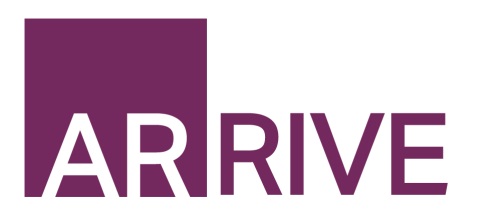


The ARRIVE Guidelines Checklist

Animal Research: Reporting In Vivo Experiments

Carol Kilkenny^1^, William J Browne^2^, Innes C Cuthill^3^, Michael Emerson^4^ and Douglas G Altman^5^

*^1^The National Centre for the Replacement, Refinement and Reduction of Animals in Research, London, UK, ^2^School of Veterinary Science, University of Bristol, Bristol, UK, ^3^School of Biological Sciences, University of Bristol, Bristol, UK, ^4^National Heart and Lung Institute, Imperial College London, UK, ^5^Centre for Statistics in Medicine, University of Oxford, Oxford, UK.*

|  | ITEM | RECOMMENDATION | Section/ Paragraph |
| --- | --- | --- | --- |
| 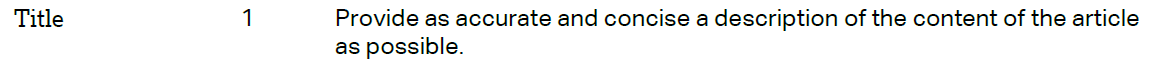 | | | Title |
| 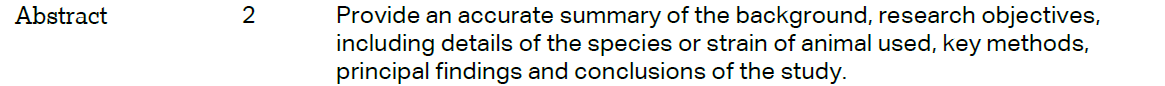 | | | Abstract |
| INTRODUCTION | | |  |
| 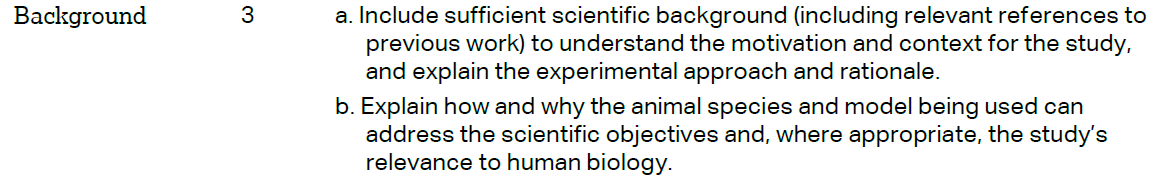 | | | Paragraphs 1-3  Paragraphs 2-3 |
| 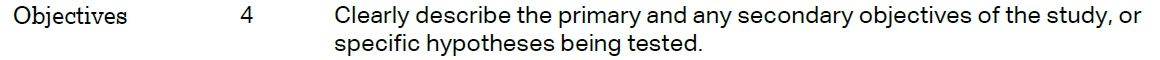 | | | Paragraph 3 |
| METHODS | | |  |
| 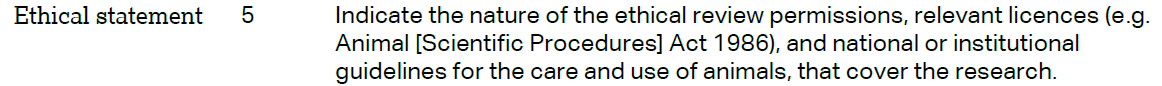 | | | Paragraph 1 |
| 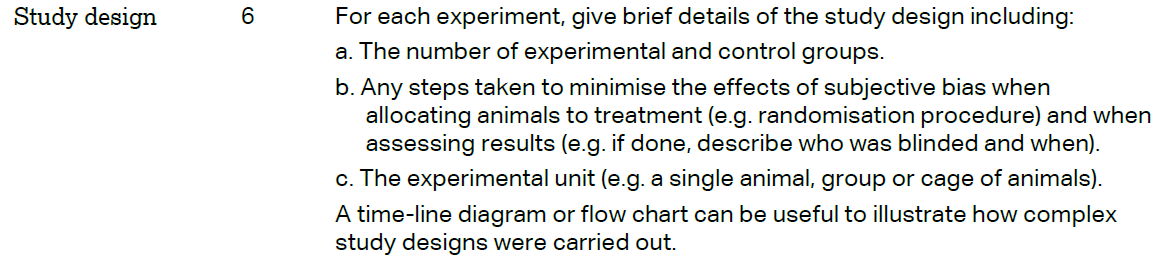 | | | Paragraph 1  Paragraph 7  Paragraph 7  Paragraph 7 |
| 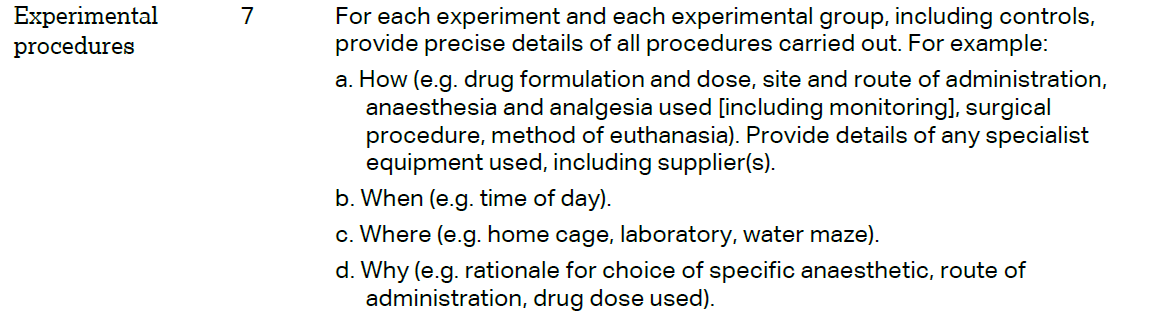 | | | Paragraphs 7 and 12 |
| 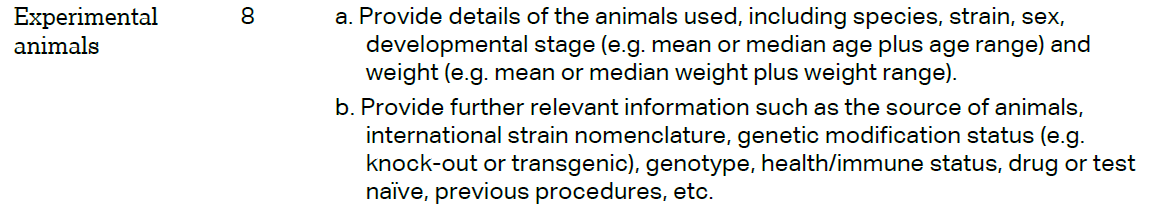 | | | Paragraph 7 |

The ARRIVE guidelines. Originally published in *PLoS Biology*, June 2010^1^

| 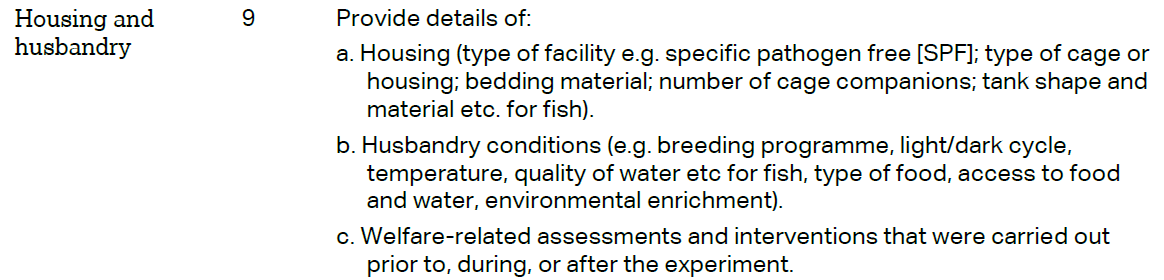 | Paragraphs 1 and 7 | |
| --- | --- | --- |
| 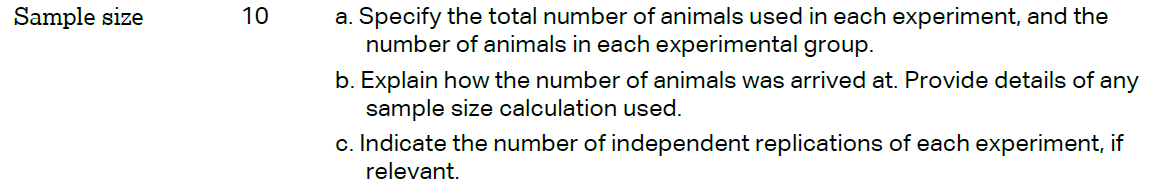 | Paragraph 7 | |
| 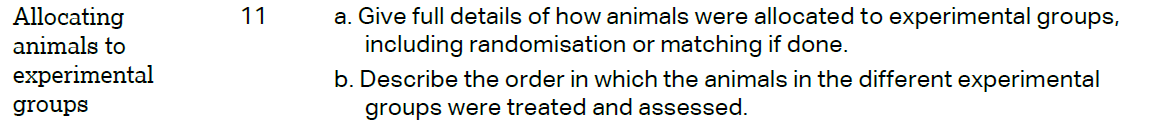 | Paragraph 7 | |
| 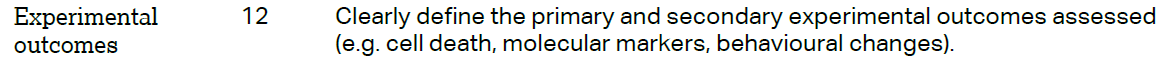 | Paragraph 7 | |
| 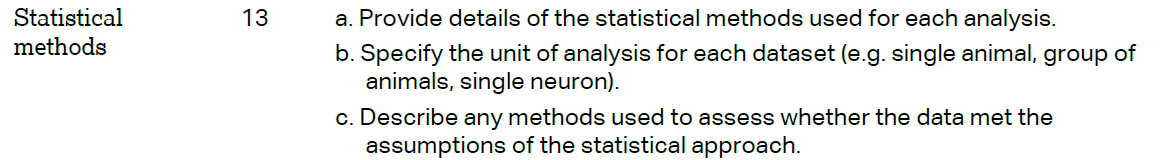 | Paragraph 13 | |
| RESULTS |  | |
| 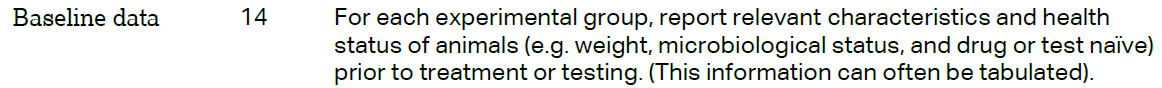 | Paragraph 5 | |
| 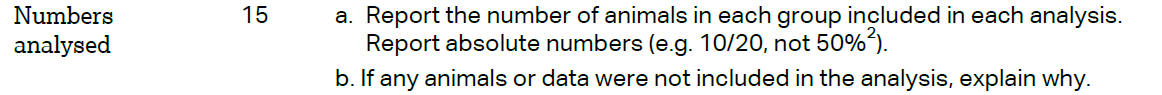 | Paragraphs 2-6 | |
| 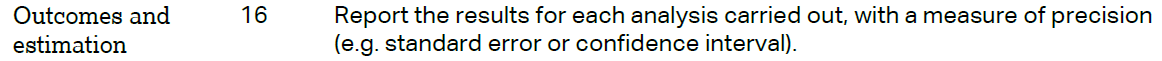 | Paragraphs 2-6 | |
| 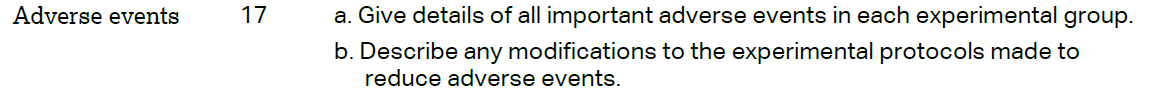 | Paragraph 5 | |
| DISCUSSION |  | |
| 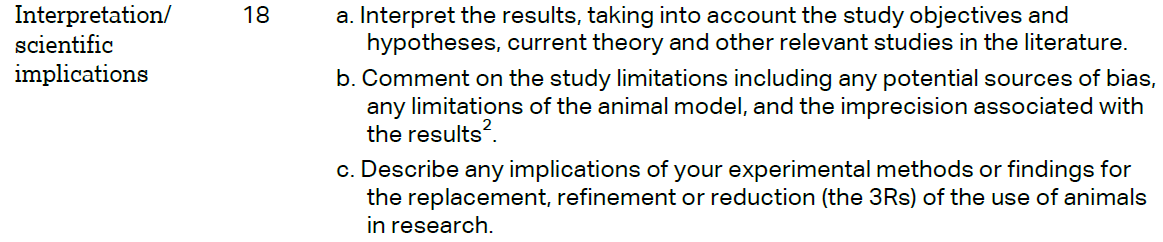 | Paragraphs 1-4  Paragraphs 1-4  Paragraphs 1-4 | |
| 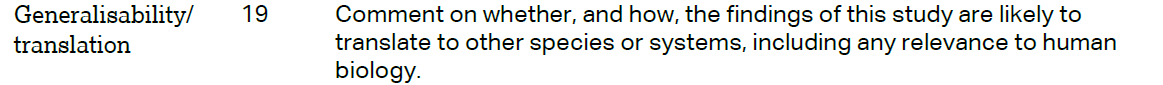 | Paragraph 1 | |
| 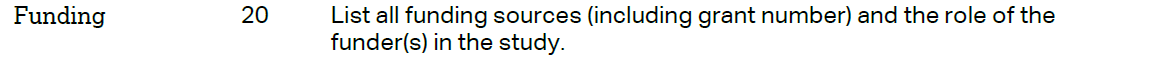 | See ‘Funding’ section on the first page |  |


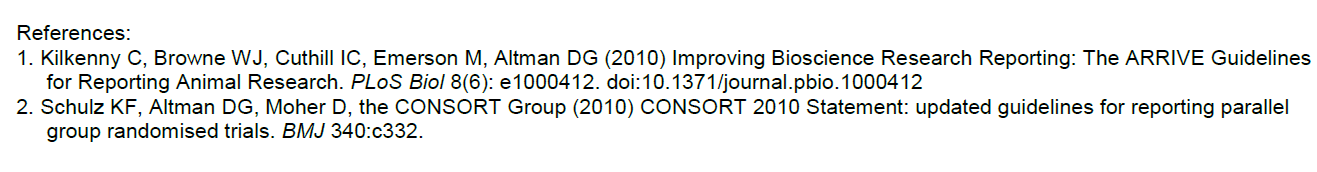

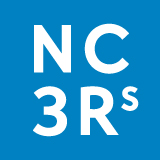

Supplement: S1 Checklist — (DOC) [file pone.0129617.s001.doc]
